# The protective effect of vitamin A palmitate eye gel on the ocular surface during general anaesthesia surgery: a randomized controlled trial

**DOI:** 10.1007/s10792-024-03074-0

**Published:** 2024-04-04

**Authors:** Siyuan Li, Guiyu Lei, Ying Liu, Lei Tian, Ying Jie, Guyan Wang

**Affiliations:** 1 Beijing Ophthalmology and Visual Sciences Key Laboratory, Beijing Tongren Eye Center, Beijing Institute of Ophthalmology, Beijing Tongren Hospital, Capital Medical University, Beijing, China; 2 Department of Anesthesiology, Beijing Tongren Hospital, Capital Medical University, Beijing, China; 3 Operating Room, Beijing Tongren Hospital, Capital Medical University, Beijing, China; 4 Beijing Advanced Innovation Center for Big Data-Based Precision Medicine, Beihang University and Capital Medical University, Beijing, China

**Keywords:** General anesthesia, Ocular surface, Postoperative dry eye, Vitamin A palmitate eye gel

## Abstract

**Purpose:**

To investigate the change in tear production associated with general anesthesia and the protective effect of vitamin A palmitate eye gel on the ocular surface during general anesthesia.

**Methods:**

This double-blind, randomized clinical trial included patients undergoing non-ophthalmic surgery under general anesthesia who randomly received vitamin A palmitate eye gel and taping for one eye (Group A, n = 60) or taping alone for the other eye (Group B, n = 60). Symptom assessment in dry eye (SANDE) score, tear film break-up time (TBUT), corneal fluorescein staining (CFS) score, and Schirmer tear test I (STT-1) were analyzed under a hand-held slit lamp before anesthesia (T_0_), 0.5 h postoperatively (T_1_), and 24 h postoperatively (T_2_).

**Results:**

At 0.5 h postoperatively, an increase in CFS score was observed in both groups (*P* < 0.05 in Group A and *P* < 0.01 in Group B), and the participants in Group A had less corneal abrasions than those in Group B. STT-1 significantly increased in Group A (*P* < 0.05), while it significantly decreased in Group B (*P* < 0.001). The changes between the two groups were statistically significant (*P* < 0.001). At 24 h postoperatively, both CFS score and STT-1 almost returned to baseline levels in the two groups. In both groups, the SANDE score and TBUT showed little change at 0.5 h and 24 h postoperatively (all *P* > 0.05).

**Conclusion:**

Vitamin A palmitate eye gel effectively protected the ocular surface and aqueous supplementation during general anesthesia.

**Trial registration:**

This study was registered in the Chinese Clinical Trial Registry (ChiCTR2100052140) on 20/10/2021.

## Introduction

General anesthesia surgery is often followed by ophthalmological complications, including dry eye disease (DED) and corneal injuries, such as corneal abrasion, recurrent corneal erosion, and exposure keratitis. According to previous studies, if no protective measures are taken during surgery, the incidence of postoperative corneal abrasion can reach up to 59% [1]. Such side effects can cause conjunctival injection, eye pain, and postoperative tearing, affecting the patient's recovery and causing serious ocular complications [2]. Therefore, ocular surface protection in general anesthesia poses a great challenge for ophthalmologists and anesthesiologists.

The most commonly used protective measure is to tape the eyelids closed. However, it has been reported that some patients complain of dry and astringent eyes after surgery, possibly due to some anesthetic auxiliary drugs' suppression of lacrimal secretion [3]. Ziyang® Vitamin A Palmitate Eye Gel (Shenyang Xingqi Pharmaceutical Co., Ltd, Shenyang, China) is a medicine that is mainly used to repair corneal epithelial cells after ocular surface damage, and as adjuvant treatment for dry eyes, meibomian gland dysfunction, and exposure keratitis. The vitamin A component is necessary for the normal differentiation of epithelial cells, and the palmitate component can effectively replenish the tear film lipid layer and reduce tear evaporation. The matrix is carbomer with high viscosity, which can enhance gel protection by physical lubrication.

The present study investigated the change in tear production associated with general anesthesia and the protective effect of vitamin A palmitate eye gel on the ocular surface during general anesthesia, including the subjective symptoms and objective signs.

## Methods

### Study design

This single-center, randomized, double-blind study included 60 participants (120 eyes) undergoing non-ophthalmic surgery under general anesthesia in Beijing Tongren Hospital in November. The study was approved by the Medical Ethics Committee of Beijing Tongren Hospital, Capital Medical University (No. TRECKY2021-137) and was registered with the Chinese Clinical Trial Registry (ChiCTR2100052140) on 20/10/2021. All methods were carried out in accordance with relevant guidelines and regulations or declaration of Helsinki. The study procedure was fully explained to the participants, who signed the informed consent.

The anesthesiologist was a specialized doctor who was also responsible for the grouping. Based on a random number table, each participant received vitamin A palmitate eye gel and taping for one eye (Group A, n = 60) or taping alone for the other eye (Group B, n = 60). The ophthalmologist was responsible for ocular examination and evaluation. Both the ophthalmologist and participants were blinded to the specific medications.

### Participants

Inclusion criteria were the following: ① age ≥ 18 years; ② patients undergoing non-ophthalmological surgery under general anesthesia of > 2 h in the supine position; ③ the scope of surgical disinfection did not involve periocular skin; ④ voluntarily participated in this study and could cooperate with the whole research process.

Exclusion criteria were: ① with active ocular diseases such as keratitis; ② with ocular surface diseases affecting outcome assessment; ③ corneal fluorescein staining (CFS) score > 3; ④ Schirmer tear test I (STT-1) < 5 mm and tear film break-up time (TBUT) < 5 s; ⑤ Robertson consciousness score < 4, unable to cooperate with the inspection; ⑥ individuals with allergies.

### Study process

All patients received general anesthesia with laryngeal mask or tracheal intubation. Anesthesia was induced with sufentanil (0.1–0.3 μg/kg), propofol (1–2 mg/kg), and rocuronium (0.6 mg/kg). Next, the anesthesiologist applied the eye gel to the conjunctival sac of the lower eyelid of the experimental eye before horizontally taping both eyelids closed. Anesthesia was maintained by continuous infusion of propofol and remifentanil, and sometimes sevoflurane alone or propofol alone or in combination, depending on the anesthesiologists' decision. Electrocardiogram, pulse oximetry, noninvasive arterial blood pressure, and end-tidal carbon dioxide concentration were monitored during the operation. The sedation level was kept within the range of 40–60 under the bispectral index (BIS) monitor. The fluctuation of mean atrial pressure (MAP) was maintained within 20% of the preoperative values of the patients. All patients received intravenous lactated Ringer’s solution at a 5–7 ml/kg rate. Atropine and neostigmine were given at the discretion of the anesthetist at the end of surgery but before extubation. The anesthesiologist removed the tape, wiped the residual gel composition around the test eye, and pushed the subject into the anesthesia recovery room.

### Measures and outcomes

The eyes were examined under a hand-held slit lamp before anesthesia (recorded as T_0_), at 0.5 h postoperatively (measured in the anesthesia recovery room, recorded as T_1_), and at 24 h postoperatively (recorded as T_2_). Examinations of all participants were arranged in the following order to avoid unexpected corneal abrasion.

Subjective symptom score: Symptom Assessment in Dry Eye (SANDE) questionnaire was used to evaluate the subjective symptoms of participants. The questionnaire included two parts based on the visual analog scale, which could quantitatively and feasibly indicate the frequency and severity of ocular surface diseases [4].

TBUT: after moistening the fluorescein sodium staining strip (Jingming New Technology Development Co., Ltd., Tianjin, China) with chloramphenicol eye drops, it was smeared on the nasal side, central side and temporal side of the lower eyelid margin. The participants were asked to close their eyes for 3 s and then open them. The tear film was observed with a cobalt blue light, and the time from the last blink to the occurrence of tear film rupture was recorded. The average value was taken three times.

CFS score: corneal abrasion was measured using cobalt blue light following TBUT evaluation. The denuded area of corneal epithelium was stained green, whereas the normal surface was left unaltered. According to the National Institutes of Ophthalmology/Industry Working Group guidelines, five corneal regions (middle, nasal, temporal, upper, and lower) were scored, with 0–3 points assigned to each region, ranging from 0 to 15 points [5].

Tear production evaluation (STT-1) was the primary outcome of this study. In the absence of ocular surface anesthesia, the filter strip (Jingming New Technology Development Co., Ltd., Tianjin, China) was placed in the temporal inferior conjunctival sac, and the participants were instructed to close their eyes; both eyes were evaluated simultaneously. The filter strip was taken out after 5 min, and the wet length was read as the result. Safety outcomes were: visual acuity, anterior segment, and facial skin were observed at each time point.

### Statistical analysis

Statistical outcomes were analyzed using SPSS22.0 (IBM Corp, Somers, NY). Median and interquartile intervals were used to describe data of ranked data (SANDE score and CFS score) and data with non-normal distribution (TBUT and STT-1). The Mann–Whitney U test was conducted to analyze the changes from baseline in the outcome measures, and the Wilcoxon rank-sum test was used for comparisons between Group A and Group B. All the hypotheses were tested as two-sided, and the *P* < 0.05 represented a statistically significant difference.

The power for the STT-1 change was calculated based on a two-sided t-test with a significance level of 5%. Using a power of 90% to detect a difference between the two groups in STT-1 and considering the probability that STT-1 would decrease to 3.6 mm/5 min in Group A and 4.3 mm/5 min in Group B with a standard deviation of 1.1, the minimum sample size required to detect a statistical difference was 52 subjects in each group. Assuming a drop-out rate of 10%, 60 eyes were enrolled in each group [6].

## Results

**Fig. 1:**
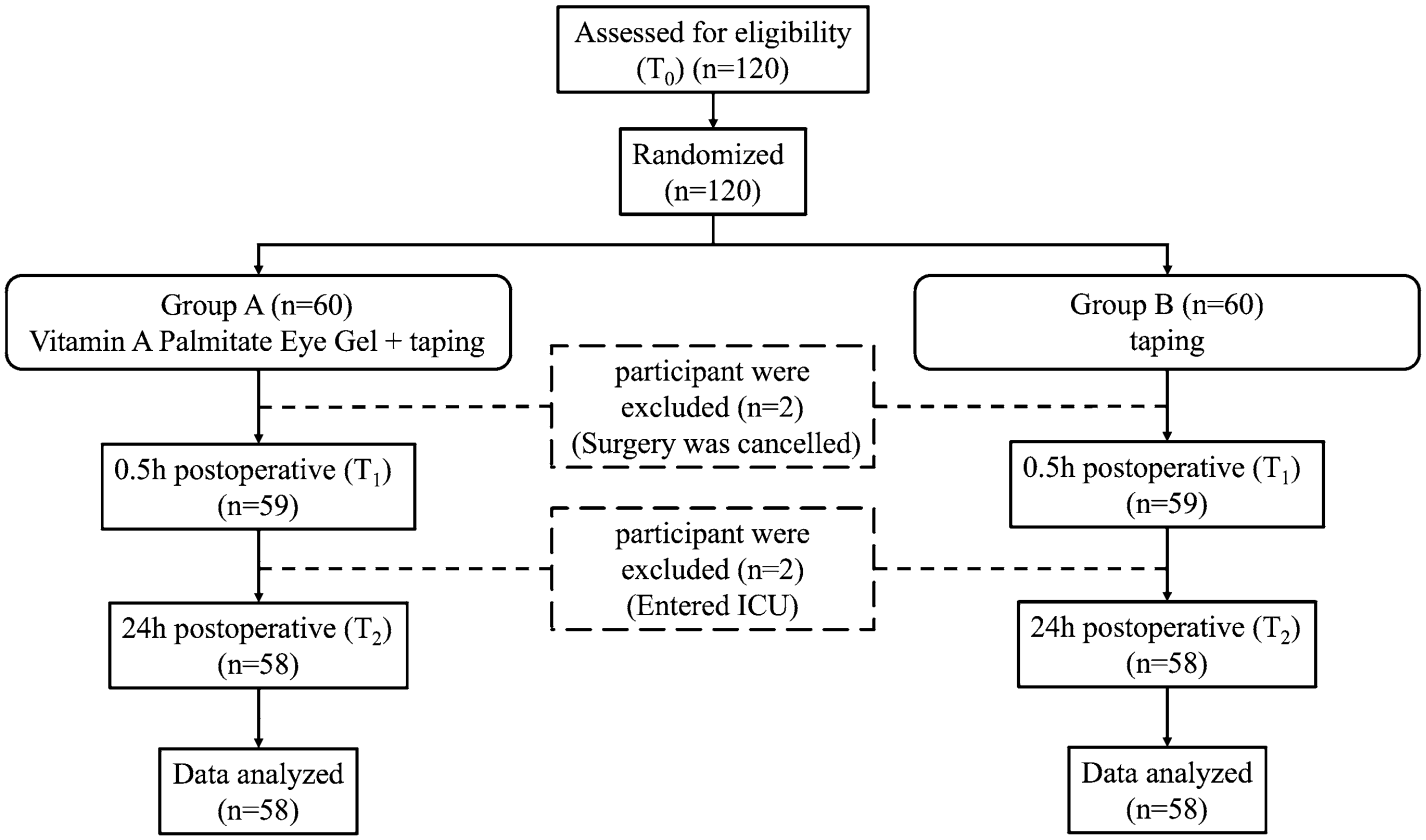
CONSORT flow diagram. ICU = Intensive care unit

Among 60 participants (120 eyes) included in this study, 58 (116 eyes) completed the entire trial process. Figure 1 shows the CONSORT flow diagram, including information about study exclusion and the sample size for analysis. Table 1 summarizes the baseline characteristics of participants in this study. The baseline levels (recorded as T_0_) are listed in Table 2. Since the study was designed as self-controlled, the data were well-matched in terms of baseline characteristics between the two groups.

**Table 1:** Characteristics of the study participants

Variable	Participants
Gender	
Female	33 (56.9%)
Male	25 (43.1%)
Age (years)	46.03 ± 11.94
BMI (kg/cm2)	23.25 ± 2.79
Smoking	
Yes	8 (13.8%)
No	50 (86.2%)
Surgery	
Thyroid surgery	41 (70.7%)
Orthopedic surgery	9 (15.5%)
Breast surgery	7 (12.1%)
Gallbladder surgery	1 (1.7%)
Operation time (mins)	179.05 ± 41.30
Operation position	
Supine	58 (100%)
ASA grade	
1	22 (37.9%)
2	36 (62.1%)

*BMI* Body mass index, *ASA* American society of anesthesiologists

**Table 2:** Satisfaction comparison between two groups, median (*P*_25_, *P*_75_)

	T_0_ (n = 120)	T_1_ (n = 116)	T_2_ (n = 116)	*PT*_*0*_*-T*_*1*_	*PT*_*0*_*-T*_*2*_
SANDE score (points)					
Group A	13.47(4.13, 20.95)	14.31(4.13, 20.95)	13.36(4.33, 20.65)	0.25	0.61
Group B	13.54(4.51, 23.86)	13.01(4.01, 20.98)	13.43(4.14, 21.27)	0.95	0.46
*P*	0.95	0.42	0.78		
TBUT (s)					
Group A	8.56(6.00, 10.29)	6.48(4.94, 8.31)	8.19(6.59, 9.49)	0.0001***	0.26
Group B	8.25(5.64, 10.54)	6.04(4.60, 6.79)	7.32(6.00, 8.61)	0.0001***	0.07
*P*	0.64	0.72	0.55		
CFS score (points)					
Group A	0(0, 1)	1(0, 1)	0(0, 0)	0.011*	0.05
Group B	0(0, 1)	1(0, 1)	0(0, 1)	0.001**	0.57
*P*	0.50	0.50	0.25		
STT-1 (mm/5 min)					
Group A	6(4, 8)	7.5(4.25, 10)	6(4, 8)	0.02*	0.68
Group B	6(3.25, 10)	4(3, 5)	6(4, 8)	0.0001***	0.14
*P*	0.91	0.0001***	0.32		
Visual acuity					
Group A	0.8(0.6, 1.0)	0.8(0.6, 0.8)	0.8(0.6, 0.8)	0.19	> 0.99
Group B	0.8(0.6, 1.0)	0.8(0.6, 0.8)	0.8(0.6, 1.0)	> 0.99	> 0.99
*P*	> 0.99	> 0.99	> 0.99		

^*^P < 0.05, **P < 0.01, ***P < 0.001

T_0_ before general anesthesia (baseline), T_1_ at postoperative 0.5 h, T_2_ at postoperative 24 h

*CFS* Corneal fluorescein staining, *SANDE*  Symptom assessment in dry eye, *STT* Schirmer tear test, *TBUT* Tear film break-up time

Compared with baseline SANDE scores, slight changes were observed at 0.5 h and 24 h postoperatively in Group A (change in scores = 14.31 and 13.36, respectively) (*P* > 0.05) and Group B (change in scores = 13.01 and 13.43, respectively) (all *P* > 0.05). There was no difference in the assessment changes at 0.5 h and 24 h postoperatively between Group A and Group B (all *P* > 0.05).

In both Group A and Group B, TBUT decreased significantly from the baseline level at 0.5 h postoperatively (change in TBUT = 6.48 s and 6.04 s, respectively) (all *P* < 0.001). At 24 h postoperatively, TBUT almost returned to baseline level in both Group A and Group B (change in TBUT = 8.19 s and 7.32 s, respectively) (all *P* > 0.05). The change trends in both groups were equivalent without significant differences (all *P* > 0.05).

At 0.5 h postoperatively, the increase in CFS score compared to baseline was statistically significant in Group A (change in scores = 1) (*P* < 0.05) and Group B (change in scores = 1) (*P* < 0.01). In direct comparison, the participants in Group A showed numerically less corneal abrasion than those in Group B. At 24 h postoperatively, there was no significant difference from baseline level in both Group A and Group B (change in scores = 1 and 1, respectively) (all *P* > 0.05).

Statistically significant improvement in STT-1 was observed at 0.5 h postoperatively in Group A (change in STT = 7.5 mm) (*P* < 0.05). However, there was a significant decrease in Group B (change in STT = 4 mm) (*P* < 0.001). The changes between the two groups were statistically significant (*P* < 0.001). At 24 h postoperatively, STT-1 almost returned to baseline level in both Group A and Group B (change in STT = 6 mm and 6 mm, respectively) (all *P* > 0.05).

**Fig. 2:**
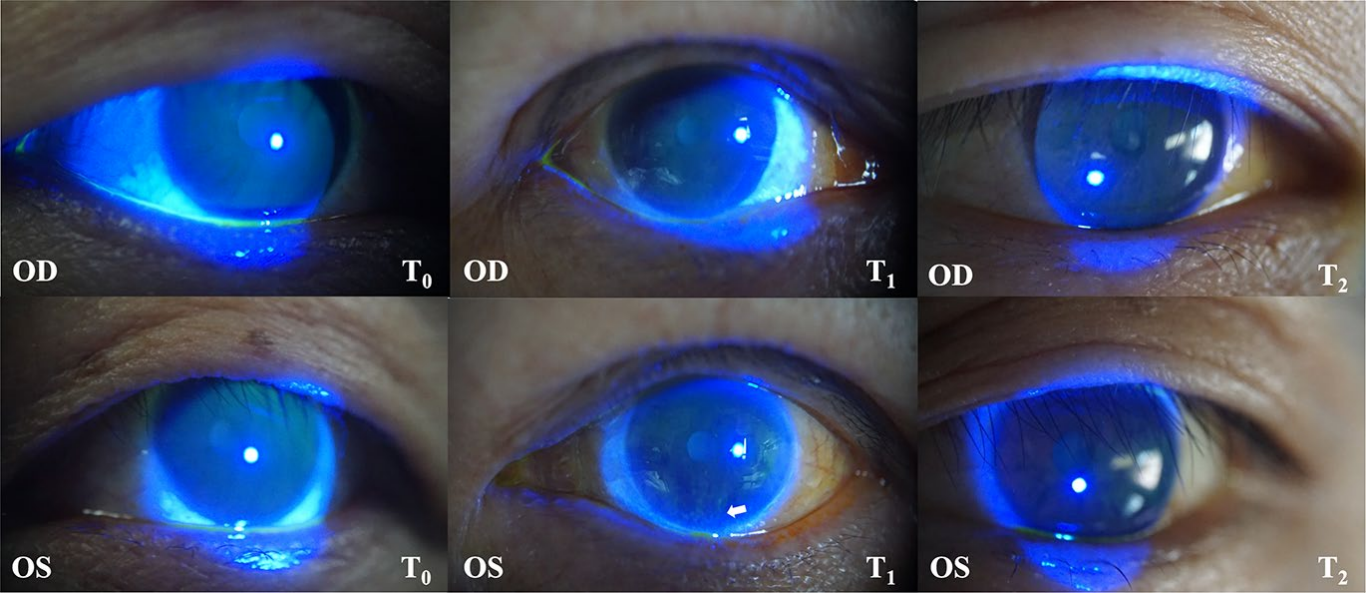
Hand-held slit lamp corneal fluorescein sodium stained cobalt blue photography

There were no significant differences observed in visual acuity across various time intervals (all *P* > 0.05), and similarly, no discernible disparities were noted between the two groups (all *P* > 0.05) Fig. 2.

The subject was a patient undergoing orthopedic surgery, and the duration of anesthesia was 197 min. The right eye conjunctival sac was smeared with VA ophthalmic gel, and the adhesive tape was transversely pasted. The left eye was simply pasted with adhesive tape. The arrow indicates the location of postoperative corneal abrasion. CFS score: T_0_ both eyes 0 points; T_1_ right eye 0 points, left eye 1 point; T_2_ both eyes 0 points.

## Discussion

Herein, we investigated the protective effect of vitamin A palmitate eye gel on the ocular surface during general anesthesia. In this randomized, double-blind self-control study, we compared the protection effect of taping eyelids alone and combined with vitamin A palmitate eye gel on each participant's subjective symptoms and objective signs. In addition to a slight increase in CFS score, the STT-1 and TBUT decreased significantly, thus suggesting that those participants suffered not only from corneal abrasions but also from postoperative DED.

DED is a multifactorial disease of the ocular surface characterized by a loss of tear film homeostasis that has a vital role in providing lubrication and protection to the cornea [7]. However, postoperative DED, following general anesthesia surgery, has not drawn much attention from the surgeons. Such patients do not necessarily experience significant pain due to no instant corneal abrasions; nonetheless, they may develop progressing DED in a few days or even weeks accompanied by discomfort and associated signs, such as decreased STT and TBUT.

Thus far, reduced tear production has been considered the main pathogenic factor of postoperative DED. Tear production is controlled by the autonomic nervous system, which is dominated by the parasympathetic system. There are G protein-coupled muscarinic receptors on the surface of lacrimal gland acinar cells and conjunctival goblet cells, which are regulated by acetylcholine (Ach). Atropine is an anticholinergic drug often used in anesthesia induction and recovery, which blocks the muscarinic receptors and reduces tear secretion. In their study, Krupin et al*.* measured the basal tear production by standardized Schirmer strips in 20 patients, finding a significant decrease at 10, 30, and 60 min following induction of the anesthesia [6]. Decreased tear production induces the deficiency of the aqueous layer.

Recent studies showed that general anesthesia may also alter tear biochemistry [8]. Zernii et al*.* reported that the development of chronic postoperative DED was accompanied by a decrease in teal film stability due to the decrease in total antioxidant activity of the tear [9]. Moreover, they reported that anesthesia induced changes in the activity of tear antioxidant enzymes, including superoxide dismutase and enzymes providing homeostasis of reduced glutathione.

Batra and Bali first addressed ocular surface protection during general anesthesia in 1977 [10]. Taping the eyelid closed, the most commonly used protection method, has certain limitations, such as inconvenient pupil observation, improper pasting, or recovery stage. Finally, removing the tape may cause mechanical damage to the ocular surface. Besides, it did not provide any ingredient supplement to the tear film.

Vitamin A, as the main component of the eye gel, has an important role in regulating epithelial growth, cell proliferation, and differentiation. According to the previous study, vitamin A upregulates secretory phospholipase in A group IIA genes in human corneal conjunctival cells, increasing mucin 16 expression [11]. In the meantime, numerous population studies showed that systemic vitamin A supplementation could improve tear quality by repopulating conjunctiva goblet cells, thus increasing their density and helping corneal re-epithelization [12–14]. Many clinical studies have shown that topical supplementation of vitamin A is efficacious in improving ocular surface conditions [15]. A study that enrolled patients with dry eyes who were unresponsive to conventional treatments found that vitamin A ointment was effective in reducing signs and symptoms and promoting goblet cell proliferation by more than 70% [16].

The palmitate component can effectively replenish the tear film lipid layer and reduce tear evaporation. The matrix is carbomer with high viscosity, which can enhance gel protection by physical lubrication.

In the current study, the STT-1 measured at 0.5 h postoperatively in Group B was significantly decreased, while that in Group A was increased (Table 2). STT is a well-standardized test that estimates basic tear secretion. The results from Group B were consistent with the existing scientific literature on general anesthesia and tear secretion. The increase of STT-1 in Group A might have occurred for several reasons. First, mucin expression promoted by vitamin A can help further increase tear film stability. Second, carbomer, as the matrix of vitamin A, is a kind of synthetic high molecular weight polymer of acrylic acid cross-linked to a polyalkenyl polyether, which forms a liquid reservoir inside the gel after acting on the ocular surface [17]. It can slowly and permanently release drugs. In addition, gel-based artificial tear supplements can offer higher viscosity and longer retention times on the ocular surface; meanwhile, they are less sticky than oil-based formulations. Thus, the aqueous composition remained sufficient for tear film in Group A after surgery, reflecting an increase of STT-1 measured at 0.5 h postoperatively.

The results showed that the CFS score measured 0.5 h postoperatively was slightly increased in the two groups. Referring to the incidence of corneal abrasion without ocular protection [10, 18], both eye gel and typing the eyelids could effectively make a difference. The CFS score in Group A was numerically lower than in Group B, which might be due to the function of vitamin A in maintaining the health of epithelial cells. TBUT in both groups decreased at 0.5 h postoperatively, revealing no significant difference between the two groups. TBUT was the most frequently employed test of ocular surface stability, and any factor that affected the composition of the tear film could lead to a decrease. According to previous studies, vitamin A palmitate eye gel can prevent the destruction of the tear film homeostasis after ocular surgery [19, 20]. Yet, only a few studies used vitamin A palmitate eye gel to prevent postoperative DED.

Furthermore, the preoperative STT-1 and TBUT were lower than the normal level; however, only two participants declared being diagnosed with DED, suggesting the characteristic of separation of symptoms and signs. Many DED patients, especially middle-aged and older people, perimenopausal people, or users of visual display terminals, would not agree to medical intervention due to the lack of obvious symptoms, even though they developed some related signs. With a healthy ocular surface environment, the reduction of tear secretion caused by general anesthesia does not cause dyshomeostasis when solely taping the eyelid closed [21]. However, if the patient already suffered from ocular surface diseases such as DED or had shown ocular surface dyshomeostasis before the surgery, in addition to the decrease in tear secretion, this could also cause a delay in lipid layer distribution and further loss of aqueous during blinking.

The current study has certain limitations. Some participants were not fully conscious when answering the questionnaire; therefore, there is a probability that the SANDE score did not accurately show their feelings. Future research will investigate the protective effectiveness of vitamin A palmitate eye gel in lengthy surgery procedures and surgeries in lateral or prone positions.

## Conclusion

Vitamin A palmitate eye gel can provide additional protection and aqueous supplement to the ocular surface during general anesthesia, especially in patients who suffer from ocular surface diseases such as DED.

## Data Availability

The data used to support the findings of this study are available from the corresponding author upon request.

## Abbreviations

CFS: Corneal fluorescein staining
STT: Schirmer tear test I
TBUT: Tear film break-up time
SANDE: Symptom assessment in dry eye
DED: Dry eye disease
ICU: Intensive care unit
BMI: Body mass index
ASA: American society of anesthesiologists
